# Seroprevalence of Equine Herpesvirus 1 (EHV-1) and Equine Herpesvirus 4 (EHV-4) in the Northern Moroccan Horse Populations

**DOI:** 10.3390/ani11102851

**Published:** 2021-09-29

**Authors:** Zineb El Brini, Ouafaa Fassi Fihri, Romain Paillot, Chafiqa Lotfi, Farid Amraoui, Hanane El Ouadi, Mohamed Dehhaoui, Barbara Colitti, Hassan Alyakine, Mohammed Piro

**Affiliations:** 1 Department of Medicine, Surgery, and Reproduction, Agronomy and Veterinary Institute Hassan II, Rabat 10000, Morocco; hassanalyakine@gmail.com (H.A.); vetpiro@yahoo.fr (M.P.); 2 Department of Microbiology, Immunology and Contagious Diseases, Agronomy and Veterinary Institute Hassan II, Rabat 10000, Morocco; fassifihri.ouafaa@gmail.com; 3 School of Equine and Veterinary Physiotherapy, Writtle University College, Lordship Road, Writtle, Chelmsford CM1 3RR, UK; romain.paillot@writtle.ac.uk; 4 Society of Veterinary Pharmaceutical and Biological Productions (Biopharma), Rabat 10000, Morocco; c.loutfi@biopharma.ma (C.L.); amraoui.farid@gmail.com (F.A.); hananelouadi@gmail.com (H.E.O.); 5 Department of Statistics and Applied Informatics, Agronomy and Veterinary Institute Hassan II, Rabat 10000, Morocco; dehhaoui@yahoo.fr; 6 Department of Veterinary Sciences, University of Turin, Largo Braccini 2, 10095 Grugliasco, Italy; barbara.colitti@unito.it

**Keywords:** EHV-1, EHV-4, seroprevalence, ELISA, VNT, Morocco

## Abstract

**Simple Summary:**

This work aims to evaluate the seroprevalence of equine EHV-1/4 in horse populations in the north of Morocco and to measure the antibody titers in vaccinated horses, under field conditions, with monovalent EHV-1 vaccines. Overall, 12.8% unvaccinated, and 21.8% vaccinated horses were positive for EHV-1. All samples were positive for EHV-4 when tested with the type-specific ELISA. The virus neutralization test showed low antibody titers in samples from vaccinated horses. Our study demonstrated that EHV-1 and EHV-4 are endemic in the horse populations in the north of Morocco and highlighted the necessity of reevaluating the vaccines and the vaccination protocol used.

**Abstract:**

This study reports the first equine herpesvirus-1 (EHV-1) and equine herpesvirus-4 (EHV-4) seroprevalence investigation in horse populations of Morocco in 24 years. It also aims to determine antibody titers in horses vaccinated under field conditions with a monovalent EHV-1 vaccine. Blood samples were collected from 405 horses, including 163 unvaccinated and 242 vaccinated animals. They were tested using a commercial type-specific enzyme-linked immunosorbent assay (ELISA) and a virus neutralization test (VNT). Overall, 12.8% unvaccinated, and 21.8% vaccinated horses were positive for EHV-1. All samples were positive for EHV-4 when tested with the type-specific ELISA. In the vaccinated group, the VNT revealed a mean antibody titer of 1:49 for EHV-1 and 1:45 for EHV-4. The present study demonstrates that EHV-1 and EHV-4 are endemic in the horse populations in the north of Morocco, with prevalence differences between regions. Furthermore, horses vaccinated with a monovalent EHV-1 vaccine had low antibodies titers. This study highlights the necessity to establish and/or support efficient biosecurity strategies based on sound management of horses and characterize further and potentially improve the efficiency of the EHV vaccines and vaccination protocol used in the field.

## 1. Introduction 

Equine herpesvirus 1 (EHV-1) and 4 (EHV-4) are common equine pathogens [1], causing significant economic losses and a negative impact on equine welfare [2]. EHV-1 and EHV-4 are closely related *Alphaherpesviruses* and, until 1981, were considered the same virus due to their genetic and antigenic similarity [3]. EHV-1 is associated with respiratory disease, abortion, neonate death, and equine herpesvirus myeloencephalopathy (EHM) [4], whereas EHV-4 is mainly related to respiratory disease, but can sporadically cause abortions [5]. The primary infection occurs through the upper respiratory tract by direct contact with respiratory secretions of actively infected horses, aborted fetuses, or placenta [6]. After the first infection, the virus establishes life-long latency (estimated to concern more than 80% of the cases), and reactivation can occur under natural conditions following transport, handling, postpartum period, or experimentally by treating horses with corticosteroids [2,7]. Consequentially, virus shedding could occur after reactivation from latency with a risk of spreading to susceptible animals.

In Morocco, the equine industry is essential for the country’s socio-economic development, with a contribution of 0.61% to the country’s GDP (Gross Domestic Product) and the direct and indirect employment of more than 30,000 people [8]. The Moroccan horse population is estimated at 110,000 horses, with around 4,000 births every year. Five main breeds are present; the Barb, the Arabian-Barb, the Arabian, Thoroughbred, and the Anglo-Arabian. The Arabian-Barb represents the majority, with 75 to 80% of the Moroccan horse population. To increase births and to reduce the losses of valuable horses, vaccination against EHV-1/4 has become a mandatory biosecurity practice required by the Moroccan authority since 2016. However, the obligation includes only breeding horses. At the same time, immunization is considered a practical approach when vaccinating a large population [9]. Moreover, vaccination efficiency in the field may vary depending on numerous factors, such as the level of virus strain circulation and/or the immune status at the time of vaccination and infection. Although EHV-1/4 vaccination reduces clinical signs of respiratory infection, virus shedding, and the occurrence of abortion storm, none of the available vaccines provide complete protection against all forms of the diseases, and none of them have been tested against EHM [5].

There is a paucity of information about the circulation of EHV-1 and EHV-4 in Morocco. The last available data come from a seroprevalence study conducted in 1997, using a virus neutralization test (VNT). This study reported an EHV-1/EHV-4 seroprevalence of 35% in tested horses [10]. Therefore, a better understanding of the EHV epidemiological situation is necessary, as it will play an essential role in preventing a disease that has a negative impact on horse welfare, breeding, and the equine sport industry. The recent EHV-1 outbreak in the CES Valencia (Spain) Spring Tours 2021 clearly illustrates the potentially devastating impact of EHV-1. Circulation of EHV-1 during this international show jumping competition that regrouped more than 750 horses has induced several hundred cases of infection, several deaths due to EHM, dissemination of the diseases in at least 9 European countries, and the subsequent cancellation of equestrian events in Europe by the FEI (Fédération Equestre Internationale) for several months (personal communication).

The development of a type-specific ELISA test, which is based on a type-specific epitope located at the C terminus of glycoprotein G (gG), represents an essential tool in the epidemiological investigation of EHV, allowing the specific sero-epizootiology and serodiagnosis of EHV-1 and EHV-4 [11]. The virus neutralization (VN) or the complement fixation (CF) tests are considered to be more cross-reactive, which tends to complicate results interpretation [12]. However, they are frequently used to assess the level of antibodies in response to a vaccination protocol. Heldens et al. [13] suggest that CF and VN antibodies may limit the duration of virus excretion, decrease the risk they pose to the other horses, and reduce the duration and severity of disease outbreaks.

The goals of this study were firstly to identify the seroprevalence of EHV-1 and EHV-4 using an EHV-1/4 type-specific ELISA in 405 sera from unvaccinated and vaccinated horses located in the provinces of Oujda, Meknes-Fez, Casablanca, El Jadida, and Marrakech. These provinces are located in the northern part of Morocco, which contains most of the horse population; and secondly to measure VN antibody titers in horses vaccinated with commercial monovalent inactivated EHV-1 vaccines currently used in Morocco, and to evaluate the serological status of the unvaccinated horses. This study is the first EHV-1/4 serological investigation conducted in Morocco in more than two decades to better understand the EHV-1/4 epidemiological situation in the north of Morocco.

## 2. Materials and Methods

### 2.1. Area and Sampled Animals

**Figure 1:**
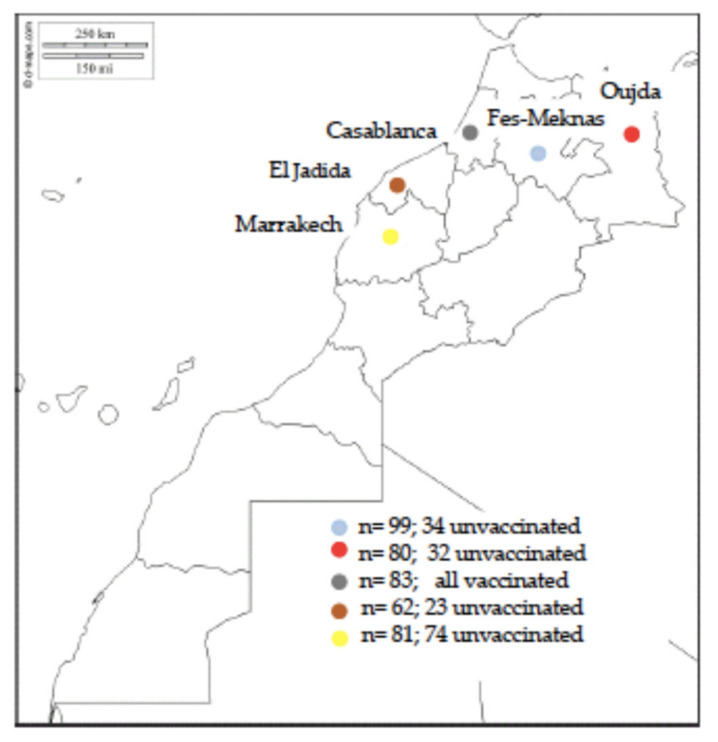
Geographical distribution of sampled regions in Morocco map.

This study was carried out on 405 horses, conveniently sampled and collected between March and May 2018, from 5 regions of Morocco that concentrate the largest population of horses in the north of Morocco and where national studs are located. The distribution of sera according to the sampling location is shown in Figure 1. 163 samples were taken from unvaccinated horses and 242 from horses vaccinated with commercially available inactivated monovalent EHV-1 vaccines. Horses were located in 5 provinces of northern Morocco (i.e., Oujda (n = 80; 32 unvaccinated), Meknes-Fez (n = 99; 34 unvaccinated), Casablanca (n = 83; all vaccinated), El Jadida (n = 62; 23 unvaccinated), and Marrakech (n = 81; 74 unvaccinated)).

The unvaccinated group was composed of 145 Arabian Barb, and 18 were from unknown breeds. There were 30% females (49/163) and 70% males (114/163) with ages ranging from 1 to 22 years (median age was 7.5 years). As the two main activities, 66% (107/163) of the unvaccinated individuals were working horses, while 34% (56/163) were horses involved in breeding.

For the vaccinated horses, 230 were Arabian Barb, and 12 were from unknown breeds. All of them were breeding horses as an activity, either stallion 20.6% (n = 50) or mares 80.2% (n = 192) with a median age of 8 years (range 4–20 years) at the time of sampling.

All immunized horses have been vaccinated by local veterinarians, under field conditions, according to the obligation of the Moroccan authorities (Royal Equestrian Society; SOREC) memorandum n° 95 of the 18/02/2015 following a preparatory course of two injections given 21 to 30 days apart. A third injection was given between 150 and 180 days (5 and 6 months) after the second injection, followed by annual boosters.

Vaccinated horses were enrolled in the study if they had received at least the primary vaccination and were vaccinated adequately with respect to the vaccination schedule. Two inactivated monovalent vaccines containing the EHV-1 Kentucky strain were used at the study time (Calvenza, Boehringer Ingelheim, Duluth, GA, USA or Pneumequine, Merial, Lyon, France) (Table 1).

All the 405 sera samples were tested for EHV-1 and EHV-4 using the type-specific ELISA.

For comparison, the EHV-1 and EHV-4 VNT were performed on samples from unvaccinated and vaccinated horses based on the ELISA results obtained (see results section):

In the unvaccinated group, the EHV-1 VNT was performed for all EHV-1 ELISA positive sera and approximately half of the negative ones. The EHV-4 VNT was conducted on a total of 36 randomly selected individuals that had previously been tested as seronegative for EHV-1 and seropositive for EHV-4 by ELISA.

Regarding the vaccinated group, the EHV-1 VNT was performed for samples from 38 randomly selected individuals that had tested positive by EHV-1 ELISA, and another 64 samples from individuals randomly selected among the negative sera defined by EHV-1 ELISA. Additionally, within the group of vaccinated horses, the EHV-4 VNT was carried out with 50 samples randomly selected from EHV-1 ELISA seronegative horses.

All the horses were clinically healthy at the time of sampling with unknown history of infection with EHV-1 and/or EHV-4. The vaccines used were commercially available and regularly registered for equine species; no suffering was caused to the animals during the blood sampling. Owners were informed, and consent to the use of blood samples in this EHV seroprevalence study were obtained.

### 2.2. Samples Preparation

Blood samples were collected by jugular venipuncture into 10 mL vacutainer tubes without coagulant (Becton Dickinson, Le Pont De Claix, France). After clotting, the samples were centrifuged at 1500 rpm for 10 min. The sera aliquots were stored at −20 °C until further processing.

### 2.3. ELISA

According to the manufacturer’s instruction, a commercial EHV-1 and EHV-4 diagnostic ELISA kit (Svanovir, Svanova AB, Uppsala, Sweden) were used to detect and discriminate EHV-1 and EHV-4 specific antibodies. The antibody values were detected by a 450 nm absorbance reading of each well. As indicated in the kit procedure, samples with optic density (OD) values > 0.20 were considered positive.

### 2.4. Virus Neutralization Test

Standard EHV VNT was performed as described in the Diagnostic Tests and Vaccines Manual for Terrestrial Animals [14]. Briefly, sera were inactivated at 56 °C for 30 min prior to the assay. The EHV-1 VNT was carried out in flat-bottomed 96 well sterile microtiter. Two-fold serial dilutions of sera were incubated with 100TCID50 per well of the EHV-1 Kentucky D strain MEM-5% FCS (Eagle’s Medium were supplemented with 5% fetal calf serum). The plates were incubated at 37 °C in a 5% CO2 atmosphere for one hour before adding 10^5^ rabbit kidney epithelial cells (RK-13, ATCC, CCL-37) in each well. The results were read microscopically after five days of culture. The highest dilution of serum resulting in 50% neutralization of virus was defined as the end-point titer. The test was validated with positive and negative serum controls and with back-titration of 100 TCID_50_ doses. Neutralizing was calculated using the Karber Spearman formula. The EHV-4 VNT was performed exactly as described above using the EHV-4 405/75 strain and Equine dermis cells (ED, ATCC, CCL-57). Titers greater than or equal to 1:4 were considered positive [15].

Virus neutralization titers are presented after log10 transformation to allow a better comparison of results.

### 2.5. Statistical Analysis

The grouping of age was based on biological criteria (taking the consideration the life expectancy of the horses, i.e., 25–30 years approximately) and also based on the age distribution of the group of horses in the study itself in order to include to the extent the possible similar number of individuals within each category. A similar approach was used for grouping time since the last vaccination and the frequency of vaccination. We assessed the number of individuals so that each category includes the possible equal number of individuals to the extent.

All statistical assumptions were checked for normality and homogeneity of variances before performing any analyses. A chi-square (X^2^) test was carried out to determine the association of the seroprevalence results with the different variables (vaccination status, regions, sex, age and activity, and the frequency of and the time of vaccination). An ANOVA test was performed to detect differences followed by a student t-test for mean comparison for multiple comparisons. Non-transformed titers were analyzed. *p* value < 0.05 was considered statistically significant. The IBM SPSS (version 25) and JMP (ver. 14.0.0) packages were used for statistical analysis.

## 3. Results

### 3.1. ELISA

A total of 405 sera samples were tested for EHV-1 and EHV-4 using the type-specific ELISA (Table S1).

All samples were found positive for the presence of the EHV-4 antibody. The EHV-1 seroprevalence was more variable. In the unvaccinated group, the EHV-1 seroprevalence was 12.9% (21/163) (95% confidence interval (CI), 8.16-19.02), whereas 21.1% (51/242) of sera collected from vaccinated horses were positive. The vaccinated and unvaccinated groups differed significantly for the EHV-1 seroprevalence response (X^2^ = 4.470, *p* = 0.0345).

A statistically significant difference was found in the unvaccinated group considering the EHV-1 antibody prevalence between different regions (X^2^ = 8.183 *p* = 0.042), El Jadida (30.4% 7/23), Fez-Meknes (5.9%, 2/34), Oujda (9.4%, 3/32), and Marrakech (12.1%, 9/74).

No significant effect was found regarding the sex (*p* = 0.730), the activity (either working or breeding horses) (*p* = 0.898), or the age (*p* = 0.256). However, in the unvaccinated horses, the incidence of the EHV-1 antibody increased significantly with age (*p* = 0.0172) (Table 2). In addition, there was no effect of the frequency (*p* = 0.718) or the time of the last vaccination (*p* = 0.075) on the seroprevalence of the EHV-1 antibody in the vaccinated horses.

### 3.2. Virus Neutralization Test

The EHV-1 and EHV-4 VNT were performed for samples from unvaccinated and vaccinated horses based on the ELISA results (Table S2) as described below:

Unvaccinated group: The results showed that 90.5% of EHV-1 ELISA positive sera were positive by VNT, with a mean antibody titer of 1:26 (4–95), while 53.6% (37/69) of the EHV-1 ELISA negative sera were positive by VNT with a mean antibody titer of 1:9 (4–24). The EHV-4 VNT revealed that 100% (36/36) of sera were positive. Their mean antibody titer was 1:19 (4–95).

Vaccinated group: The mean antibody titer was 1:49 (8–219) for EHV-1 positive and negative EHV-1 ELISA sera (no significant differences in their mean antibody titer (*p* = 0.78)) and 1:45 (8–166) for EHV-4. No significant difference in the mean antibody titer was found between EHV-1 and EHV-4 titers (*p* = 0.51). The ANOVA showed no effect of age and the frequency of vaccination on the values of the VNT. However, there was a significant effect on VNT values for the number of days since the last vaccination. VN values decreased when the number of days since the previous vaccination increased (Table 3). All sera from vaccinated horses were positive (titers > 1:4) for EHV-1 and EHV-4.

## 4. Discussion

This study represents the first EHV-1 and the EHV-4 seroprevalence investigation conducted in the Moroccan horse populations in the last 24 years. Samples were collected from both vaccinated and unvaccinated, working and breeding horses located in five different regions of the north of Morocco. Serum was analyzed with the type-specific ELISA and the EHV-1/4 VNT.

### 4.1. Type-Specific ELISA

This study showed an overall EHV-1 seroprevalence rate of 12.8% in unvaccinated horses, while 100% of samples were positive for EHV-4. The high EHV-4 seroprevalence could be explained by an endemic circulation of EHV-4 with recurrent infection during the horse lifetime, inducing the antibody response to reach a plateau level [16]. EHV-4 outbreaks can occur all year round, with no link to seasonal variations, whereas EHV-1 outbreaks are usually reported in winter or early spring [17,18]. Moreover, Crabb et al. [19] suggest that the reactivation and/or reinfection with EHV-1 is less common. Consequently, the antibody response probably declines over time.

Interestingly, the EHV-1 seroprevalence in the vaccinated group was only 21.1% (51/242), regardless of time since or the frequency of vaccination. Despite that, all horses have received at least a primary course of vaccination. Our results suggest that the commercial type-specific ELISA could not reliably detect the antibody response produced by the EHV-1 vaccines used in Morocco. A study conducted by Yasunaga et al. [20,21] reported no difference in the antibody titer using a gG ELISA compared with the CF that revealed a significant increase in antibody titer after repeated intramuscular or intranasal vaccinations with an inactivated EHV-1 vaccine. In contrast, the study from Crabb et al. [19] reported that the type-specific ELISA was sensitive enough to detect a gG-specific antibody response after vaccination with an inactivated EHV-1/4 vaccine. The sensitivity of the gG ELISA might explain the difference. Crabb et al. [19] used a serum dilution of 1/1000, while the one used in the current study required a dilution of 1/10,000. The 100% seropositivity for EHV-4, in the vaccinated horses is likely to represent the seroprevalence of EHV-4 infection in the northern Moroccan horse population. However, in the absence of an EHV vaccine with DIVA capacity (Differentiating Infected from Vaccinated Animals), it is difficult to conclude if the current study’s seropositive results obtained with the gG ELISA are linked to vaccination and/or natural infection.

There was a significant difference between the EHV-1 seroprevalence in the unvaccinated group (12.9%) when compared with the vaccinated populations (21.1%) by the ELISA test. Statistical analyses showed no effect of the frequency or the time since the last vaccination on the seroprevalence of the EHV-1. While not statistically significant in the current study, the horse sex may need to be considered. The majority of the vaccinated horses were mares (80.2%, 194/242), with breeding as the main activity (90.5%, 219/242), while the unvaccinated ones were primarily working horses (62%, 101/163) and male (69.9%, 114/163). It has been demonstrated that breeding mares are the principal reservoir of EHV-1 [22]. They undergo significant stress around the breeding and weaning period, resulting in more frequent reactivation of latent infections [23,24].

Numerous epidemiological investigations have been performed to measure EHV-1/4 seroprevalence worldwide. In our study, the overall EHV-1 and EHV-4 seroprevalences were 12.9% and 100%, respectively. In Morocco, previous studies have reported a seroprevalence between 32.38% and 51.5% for EHV-1 using VN and CF tests [10,25,26,27]. The strong cross-reaction might explain the difference in seroprevalence between EHV-1 and EHV-4 [12]. While similar seroprevalence using the same type-specific ELISA for EHV-1 18.8, 30, 23.2, and 21.1% and EHV-4 98.7, 100, 78, and 100% were reported, respectively by Dunowska et al. (New Zealand) [28], Ataseven et al. (Turkey) [29], Sáen et al. (Colombia) [30], and Crabb et al. (Australia) [19]. In a study conducted in Israel, a similar seroprevalence (99%) to EHV-4 was reported, with a very low seroprevalence (1%) to EHV-1 [31].

The results of the current study show an essential variation between regions. The higher EHV-1 seroprevalence was observed in El Jadida. This region encompasses the largest number of equids; breeding activity/farms, commingling, competition (racing, fantasia), and transportation of horses. These factors were identified as significant risks for the circulation of EHV-1 in horses [24]. When compared with another study [32], no climatic effect was associated with the regional seroprevalence of EHV-1 as all studied regions have a Mediterranean climate with only slight seasonal variations. Moreover, the lowest seroprevalence was in regions characterized by a cold winter, which has been identified as a stressor factor for EHV reactivation [17,33]. According to some studies, an increased incidence of EHV-1 seropositivity was observed in relation to the age in the unvaccinated horses [32,34]. Paillot et al. [35] reported that cell-mediated immunity to EHV-1 increased with age, which could be linked to repeated reactivation of latent EHV-1, infection, and vaccination. This result was not observed in the vaccinated horses. The effect of the vaccination might explain this difference, with the EHV-1 vaccine administered at an early age, potential frequent vaccination, and impact on infection/re-infection. However, this hypothesis needs to be confirmed in a more significant number of horses.

In contrast to the current study, the study conducted in Morocco by Hmidouch et al. in 1997 [10] found no effect of the horse density on the geographical distribution of the EHV-1/4 prevalence. The highest prevalence was observed in the region of Marrakech (39.07%), while the lowest prevalence was reported in the regions of El Jadida–Casablanca (24.93%). On the other hand, similar to our results, the sex and the age of the animal had no impact on the seroprevalence of the EHV1/4.

### 4.2. Virus Neutralization and ELISA Test

The neutralization antibody titers measured against EHV-1/4 in unvaccinated horses support a previous exposure and an active circulation of these viruses in horse populations of the north of Morocco. This result highlights the importance of this group as a source of infection and contamination for naive horses. We also found that the samples found negative when tested with the EHV-1 ELISA were, in fact, positive when tested with the VNT. Considering the high sensitivity and specificity of the type-specific ELISA as reported in previous studies [12,15,19], this result may be explained by the cross-reactivity between the EHV-1 and EHV-4 due to their antigenic similarity. In contrast, the EHV-4 VNT shows that all sera were positive in accordance with EHV-4 ELISA. These results strongly prove that EHV-4 is a ubiquitous virus actively circulating in horse populations in the north of Morocco.

In vaccinated horses, the aim of the VN assay was mainly to evaluate the antibody titers induced by an EHV-1 monovalent vaccine administered in field conditions. Our mean antibody titer was 1:49 for the EHV-1 and 1:45 for the EHV-4. Direct comparisons with other studies cannot be easily made, as our means were calculated on horses that were vaccinated on different days of the schedule of the vaccination program. However, relying only on the time since the last vaccination, even in the group that was vaccinated less than 90 days before sampling, the antibody titers remain low in comparison to other studies using an inactivated EHV vaccine (1:137-2048) [13,36,37] or the modified live (1:115-2048) [36,37]. This difference can be related to different factors. This is mainly due to the difference in the type of the vaccines, vaccination schedules, and the vaccine status at the time of vaccination. Indeed, Bannai et al. [38] suggest an effect of the previous infection with the EHV-4, which is antigenically cross-reactive with EHV-1 and could limit the increase in the antibody titer following vaccination. Attili et al. [39] suggested that the vaccine administration in animals with high antibody titers due to infection or previous vaccination could induce a decrease in antibody titer due to an interaction between antibodies and the vaccine. Based on the ELISA results, all our horses were positive to EHV-4.

There was no measurable effect of age or the frequency of the vaccination on the levels of the antibody titers. However, there was an inversely proportional relationship between the time of the vaccination and the VN antibodies titer; the fewer days between the time of vaccination and the sampling, the higher the VN values. This result was also reported in other studies, where the antibody titer started to decline 3 to 6 months after the vaccination [40,41,42]. Consequently, the approved vaccination protocol in Morocco may need to be reevaluated in order to incorporate more regular boost immunization for better protection.

In Morocco, horses were vaccinated with a monovalent EHV-1 vaccine to gain immunity for both viruses based on their genetic similarity. However, Lang et al. [15] revealed that even in natural infection, the increase in antibodies to the other virus was insufficient to generate a considerable seroconversion as the complete DNA sequence has proven significant genetic differences between the two Alphaherpesvirus [43,44]. The results of our study revealed no statistically significant difference in the average antibody titer against both viruses. This result may partly be explained by detecting cross-reactive antibodies when using the VNT, as previously demonstrated by Hartley et al. [12]. Moreover, Heldens et al. [13] suggested that monovalent vaccines would not offer adequate protection against heterologous challenges, and it would be unwise to rely on cross-protection. As a consequence, the use of the monovalent EHV-1 vaccine in Morocco may need to be reevaluated.

Finally, it is worth mentioning that the Arabian barb breed was overrepresented in the population sampled (i.e., 90.4%; due to the sampling process, availability, and selection) when compared with the overall Moroccan horse breed distribution (75 to 80%). Another potential limitation of the study is the imbalanced number between the group of vaccinated and unvaccinated in each region and the limited number of horses tested by the VNT. Therefore, our study only provides a snapshot of the situation and may not entirely represent Morocco’s horse population.

## 5. Conclusions

EHV-1 and EHV-4 are endemic in horse populations in the north of Morocco. The EHV-1/4 type-specific ELISA revealed that all the horses were seropositive to EHV-4, while the seroprevalence of EHV-1 was more related to the region of origin. On the other hand, our results demonstrated that horses vaccinated in field conditions with a monovalent inactivated EHV-1 have a low level of antibody titers. An inversely proportional relationship was observed between the time since the last vaccination and the VN antibody titer. Considering these results, and the low frequency of vaccinated horses with measurable antibody titers, the vaccine and/or the vaccination schedule may need to be reevaluated. Epidemiology studies looking at the prevalence of EHV-1 and EHV-4 infection in Morocco will be necessary to confirm the level of EHV circulation and protection induced by vaccination. Moreover, further investigations will also be required to determine the annual losses due to EHV-1/4 in Morocco.

## Figures and Tables

**Table 1:** Information related to different vaccination parameters for the 242 vaccinated horses included in the study.

Vaccination Parameter	Category	Number of Individuals
Vaccination frequency	2 times	101
	3 times	81
	≥4 times	60
Days since last vaccination	1–90	85
	91–180	98
	≥181	59
Vaccine type *	A	170
	B	38
	C ^#^	34

* A: Calvenza, B: Pneumequin, and C: Pneumequin and Calvenza ^#^ (^#^ last vaccine used in 93% of the horses).

**Table 2:** The difference for EHV-1 antibody values by the ELISA type-specific based on sex, activity, and age group of the horses included in the study.

		All Population		Non-Vaccinated		Vaccinated	
Variable	Df	X^2^	*p*	X^2^	*p*	X^2^	*p*
Sex	1	0.552	0.4576	0.740	0.3897	1.300	0.2542
Activity	1	0.073	0.7876	2.070	0.1502	1.339	0.2472
Group of age	2	1.928	0.3814	8.125 *	0.0172 *	2.615	0.2705

* Significance for X^2^ and *p* value. X^2^: chi-square, DF: degrees of freedom for treatments, and *p*: probability.

**Table 3:** Mean comparison of VNT dependent on the age, vaccination frequency, and the time since the last vaccination for the EHV-1 and EHV-4 combined.

Vaccination Parameter	Category	Number of Individuals	VN
Age (years)	1–6	63	44
	7–10	72	49
	≥11	24	52
Vaccination frequency	2 times	78	51
	3 times	59	45
	≥4 times	22	47
Days since last vaccination	1–90	73	56a
	91–180	59	42b
	≥181	27	36b

Means accompanied by different letters under the same column differ significantly for α = 0.01.

## Data Availability

The data presented in this study are available in Tables S1 and S2.

## Supplementary Materials

The following are available online at https://www.mdpi.com/article/10.3390/ani11102851/s1, Table S1: Data—ELISA EHV Morocco, Table S2: Data—VNT EHV Morocco.
